# Parent Education and Future Transition to Cigarette Smoking: Latinos' Diminished Returns

**DOI:** 10.3389/fped.2020.00457

**Published:** 2020-08-19

**Authors:** Shervin Assari, Shanika Boyce, Cleopatra H. Caldwell, Mohsen Bazargan

**Affiliations:** ^1^College of Medicine, Charles R Drew University of Medicine and Science, Los Angeles, CA, United States; ^2^Department of Health Behavior and Health Education, University of Michigan School of Public Health, Ann Arbor, MI, United States; ^3^Department of Family Medicine, University of California, Los Angeles, Los Angeles, CA, United States

**Keywords:** education, smoking, tobacco use, ethnicity, socioeconomic status, youth

## Abstract

**Background:** High parent education is protective against youth health risk behaviors such as tobacco use. According to the *Minorities' Diminished Returns* theory, however, higher parent education seems to exert less protection for the ethnic minority relative to the majority groups.

**Objectives:** To explore ethnic differences in the effects of parent education on the transition to cigarette smoking in a national sample of American never-smoker adolescents.

**Methods:** This longitudinal study used data of waves 1 and 4 of the Population Assessment of Tobacco and Health (PATH 2013–2018). This analysis included 5,021 American youth who were never smokers at baseline (2013) and were followed for 4 years. Transition to cigarette smoking was the dependent variable. Parent education was the independent variable. Youth age, youth gender, and family structure were the covariates. Ethnicity was the moderating variable.

**Results:** From the 5,021 American youth who were never smokers at baseline (2013), 89.4% continued as never smokers, and 10.6% became ever-smokers. Overall, 4.0% were current smokers at wave 4. Overall, a higher parent education was associated with lower odds of transitioning to ever and current cigarette smoker at the end of the 4th year. Parent education, however, showed significant interaction with Latino ethnicity on both outcomes suggesting smaller protective effects of high parent education against transitioning to tobacco use for Latino than for non-Latino youth.

**Conclusions:** In the U.S., ethnicity alters the magnitude of the protective effect of parent education against youth transition to tobacco use. While high parent education is protective against transitioning to become a cigarette smoking overall, non-Latinos (a socially privileged group) gain more and Latino youth (a socially marginalized group) gain least from such a resource. In addition to addressing the SES gap, policymakers should identify and address mechanisms by which ethnic minority youth remain at risk of tobacco use, even when they are from highly educated families.

## Background

Tobacco is still among the leading preventable causes of disease in the US (1–3). About 480,000 Americans die from tobacco-related illnesses annually. Besides, more than 16 million Americans are impacted by diseases caused by tobacco (4). These tobacco-related illnesses cost the U.S. more than $300 billion each year. Unfortunately, there is an unequal health burden from tobacco use in the U.S (4). Despite the progress in reducing overall morbidity and mortality attributed to smoking, tobacco use has shifted from a mainstream public health issue to a concentrated public health problem predominantly impacting marginalized populations defined by socioeconomic status (SES) and ethnicity (5).

In line with the Minorities' Diminished Returns (MDRs), (6, 7) refer to “*less than expected”* health effects of family socioeconomic status, particularly parent education in ethnic minority groups compared to ethnic majority youth (8–11). These patterns are persistent across SES resources and outcomes 12. They suggest that (a) not all ethnic disparities are caused by SES gaps but also by differential health gains from social and economic resources for ethnic groups compared to Whites; (b) relatively speaking, ethnic gaps in a wide range of outcomes may increase rather than decrease as SES levels increase; and (c) we should address ethnic disparities across all SES spectrum, rather than merely focusing on disparities in the low SES ethnic minorities (6, 7). The result of this line of work redirects the attention from focusing on health disparities in low SES (12) to middle-class ethnic minorities (13–16), which is a growing section of the U.S. demography. This view is also similar to what Navarro has proposed as ethnicity “and” SES rather than ethnicity “or” SES as the primary contributor to health disparities (17–19). Finally, it emphasizes the lack of comparability of SES across ethnic groups (20).

The MDRs have been applied to explain ethnic differences in tobacco (21, 22) and alcohol (23) use in adolescents (24) adults (21, 22, 25). Among adults, a study using a national random sample of Whites and African Americans documented a larger effect of educational attainment on alcohol use among Whites than among African Americans (23). In a study using the National Survey of American Life (NSAL), educational attainment showed a smaller protective effect on smoking among African American adults than among White adults (21). In the HINTS data that are also nationally representative of adults, education showed a larger effect on reducing tobacco use of Whites than African Americans (21, 26). In another study of adults that used a representative sample of Los Angeles residents, employment had a larger protective effect against smoking for non-Latino than for Latino adults 23. In the only published study on MDRs of parent education in adolescents, using cross-sectional data of the baseline of Population Assessment of Tobacco and Health (PATH 2013) showing parent education better reduces tobacco dependence in Whites than African Americans and Latinos (24). In another cross-sectional study that used the PATH-Adults data, educational attainment better reduced hookah smoking for Whites and non-Latinos than African Americans and Latinos (27). In a PATH-Adults study, high education was associated with a larger increase in tobacco harm knowledge in Whites and non-Latinos than African Americans and Latinos (28). In the PATH-Adults study, education better reduced exposure to tobacco ads for non-Latino Whites than African Americans and Latinos (29). In another study using the PATH-Adults study, education better reduced the risk of smoking in non-Latino Whites than in Chinese Americans (30). In the National Health Interview Survey (NHIS) study, educational attainment showed larger protective effects against second-hand exposure to tobacco smoke in home (31) and workplace (32), for Whites than for African Americans. None of these studies, however, have used longitudinal data.

Extensive research has suggested that MDRs also apply to youth. MDRs for youth are not specific to tobacco use, as they hold for a wide range of outcomes (8–11). Parental education is associated with lower obesity (11, 33), chronic disease (24, 34–36), aggression (24), tobacco use (24), and impulsivity (9) for Whites than for African Americans. Similarly, parental education better boosts school bonding (37) and school performance (24, 38–40) of White than African American youth. Parental education generates less tangible health outcomes for African American youth than for non-Latino White youth (9–11, 24, 40–42). However, for at least two reasons, there is a need to conduct more studies on the contribution of MDRs to tobacco health disparities in Latino youth. First, most of the MDR literature is on African American rather than Latino youth (9–11, 24, 40–42). Very few studies have ever shown such patterns for Latino youth. Second, almost all of the existing literature on MDRs is using cross-sectional data, with almost no studies testing ethnic variation in the protective effects of education on future transition in tobacco use. Thus, there is a need to extend this literature to longitudinal studies that compare the protective effects of parent education for Latino and non-Latino adolescents over time.

For several reasons, ethnic minority youth, particularly Latino youth, are a high-risk group smoking in the US (43). Latino white (vs. non-Latino white) youth may perceive tobacco products as less dangerous (44). Latino white youth are more likely to experiment with tobacco products than non-Latino youth are (45–48). Latino youth may have lower perceived harm of e-cigarettes relative to non-Latino white youth, which may increase their risk of initiation, experimentation, and continuation (49). Non-Latino white youth are experiencing a faster decline in smoking rates than ethnic minorities are, including Latino youth (50, 51). Latinos have reduced access to smoking cessation treatments. Latinos are also less likely to receive advice to quit from their healthcare professionals. On top of discrimination and bias, this is in part because Latinos have less healthcare access and lower health insurance coverage. Finally, Latinos are also more likely to relapse following smoking cessation attempts relative to non-Latino whites (43). Latino youth are at a higher risk of transitioning to more stable use patterns than White youth. This is shown as a potential increased risk of transition from experimental to regular use in Latino youth. Latinos with cigarette smoking may be less likely to have successful cessation. These observations are suggestive of a telescoping effect of tobacco use in Latino youth and other ethnic minorities. As a result of such vulnerability, despite a later smoking onset, Latino youth are more likely to show undesired tobacco outcomes and tobacco-related illnesses and chronic diseases (43, 45–49).

### Aim

Using the longitudinal data of PATH that followed a nationally representative sample of U.S. adolescents for four years from 2013 to 2018, we performed this study to explore whether adolescents' ethnicity alters the effects of parent education on change in tobacco use of non-smoker adolescents over time. We hypothesized that the protective effect of parent education against transitioning to tobacco use would be smaller for Latino than in Non-Latino White youth.

## Methods

### Design and Settings

This longitudinal study is a secondary analysis of waves 1 and 4 of the PATH–Adolescents. PATH is the main longitudinal study of tobacco use in the U.S. Wave 1 and wave 4 of the data were collected in 2013–2014 and 2017–2018, respectively.

### Sample and Sampling

The PATH study's adolescent sample in Wave 1 was the civilian, non-institutionalized, U.S. population 12–17 years old in the U.S. The current analysis was limited to the 5,021 adolescents who were never-smokers at baseline and were followed for 4 years and had valid data on their smoking status at wave 4. The PATH study used a four-stage stratified area probability sample design to recruit participants. Using stratified sampling, at the 1st stage, 156 primary sampling units (PSUs) were selected. The PATH geographical PSUs were composed of single or a group of counties. The 2nd stage was formed from sampled smaller geographical segments in each PSU. The 3rd stage sampled residential addresses. The 4th stage was the selection of one adolescent and adult participant within households. Participants completed a questionnaire using an Audio Computer-Assisted Self-Interview system.

### Study Variables

The study variables included adolescent age, gender, ethnicity, parent education, parental marital status, all measured at wave 1, and tobacco use at wave 4.

#### Dependent Variables

##### Ever cigarette smoking

The primary outcome was transitioning to ever cigarette smoking. Ever use was measured using the following question: “Did you ever try cigarette smoking, even one or two puffs?”(1=Yes, 0=No).

##### Current cigarette smoking

The secondary outcome was transitioning to current smoking. Current cigarette smoking at wave four was measured by a series of items, including asking participants whether they smoked in their life, in the past 30 days, and how much they smoked. Those who said they had smoked were asked about the number of days they smoked during the past 30 days question. So, we could identify individuals who were current smokers (ever smoker + smoked over the past 30 days). Responses on this item were 0 and 1.

#### Independent Variable

##### Parent education

Parent education was measured as a categorical variable with three levels: (1) less than high school graduation, (2) high school graduation, and (3) college graduation.

#### Moderator Variables

##### Ethnicity

Adolescent ethnicity was used as moderator variable and was operationalized as dichotomous variable: African American vs. White and Latino vs. Non-Latino.

#### Demographic Confounders

Age, gender, and family structure were the covariates. Age was measured as a dichotomous variable: (1) 12–15 years old and (2) 16–17 years old. Gender was a dichotomous variable. Family structure was a dichotomous variable; 1= married, 0 = otherwise.

#### Data Analytical Plan

As we were interested in the longitudinal association between parent education and subsequent tobacco use outcomes at wave 4, our predictors were all measured at wave 1 (baseline), and the outcome was measured at wave 4. Since the PATH study uses a complex multistage sampling design and provides survey weights to makes results generalizable to the U.S. population, we applied the weights and used Taylor series linearization for variance estimation. We first explored the separate and joint distribution of our variables. We ruled out collinearity between our variables (ethnicity, educational attainment, and parental marital status). For univariate analysis, we used a frequency table. Next, we used multivariable logistic regression analysis with binary tobacco use measures as outcomes. We ran two models in the pooled samples for each outcome. *Model 1* had no interaction terms. *Model 2* always had two interaction terms between ethnicity and parent education. For the main effects of parental education on smoking outcomes, ORs <1.00 are protective. For race by education interaction terms, OR <1.00 is indicative of larger protection for African Americans or Latinos than Whites and non-Latinos. We used SPSS 23.0 (IBM Corporation, Armonk, NY, USA) to analyze the data.

#### Ethics

All adolescent participants provided written assent. When they became adults, they provided consent. All the parents or guardians also provided permission and consent to be interviewed. The study protocol was revived and approved by the Westat Institutional Review Board. We, however, used the PATH public data set, which is fully de-identified. As a result, the current analysis was non-human research and was exempt from an IRB review.

## Results

### Descriptive Statistics

This study included 5,021 American youth who were never smokers at baseline (2013). From this number, 4490 (89.4%) continued as never smoker, and 531(10.6%) became ever-smoker. Table 1 shows the descriptive data in the total sample.

**Table 1:** Descriptive statistics (*n* = 5,021).

	***n***	**%**
**ETHNICITY**		
Non-Latino	3,414	69.7
Latino	1,484	30.3
**ETHNICITY**		
White	3,210	79.8
African American	812	20.2
**AGE**		
12–15	4,853	96.7
16–17	168	3.3
Gender		
Women	2,460	49.2
Men	2,543	50.8
Family structure		
Non-married	1,737	34.7
Married	3,268	65.3
**PARENT EDUCATION**		
Less than high school graduation	1,018	20.4
High school graduation	2,414	48.4
College graduation	1,559	31.2
**LIFETIME (EVER) CIGARETTE SMOKING**		
No	4,490	89.4
Yes	531	10.6
**CURRENT SMOKING (PAST 30-DAY)**		
No	4,818	96.0
Yes	201	4.0

*Percentages are weighted*.

### Multivariable Models

Table 2 shows the results of two nested logistic regression models with parent education as the independent variable and tobacco use outcomes as the dependent variable. Both models were estimated in the pooled sample. While *Model 1* only included the main effects of parent education, ethnicity, and covariates, *Model 2* also included interactions between ethnicity and parent education.

**Table 2:** Logistic regressions on the transition to ever cigarette smoker in the pooled sample.

	**Model 1**		**Model 2**	
	**Main effects**		**Main effects** **+** **interactions**	
	OR	95% CI	OR	95% CI
Ethnicity (Latino)	0.84	0.65–1.08	0.47^**^	0.29–0.78
Ethnicity (African American)	0.39^***^	0.28–0.54	0.37^**^	0.19–0.70
Gender (male)	1.10	0.89–1.34	1.10	0.90–1.35
Age (18–21)	1.41	0.86–2.33	1.43	0.87–2.36
Parents married	0.62^***^	0.50–0.77	0.63^***^	0.51–0.78
Parent education				
Less than high school graduation	1.00		1.00	
High school graduation	1.08	0.82–1.43	0.79	0.53–1.18
College graduation	0.65^*^	0.46–0.90	0.45^***^	0.30–0.70
High school graduation × Latino			2.04^*^	1.13–3.68
College graduation × Latino			2.86^**^	1.30–6.31
High school graduation x African American			0.97	0.46–2.07
College graduation × African American			1.30	0.47–3.58
Intercept	0.20^***^		0.27^***^	

* *p < 0.05*,

** *p < 0.01*,

*** *p < 0.001*.

*CI: confidence interval; OR: odds ratio*.

Based on *Model 1*, high parent education was associated with lower odds of transition to ever smoker status. *Model 2* showed interactions between parent education and Latino ethnicity on the transition to ever smoker status, which suggests that a higher parent education has a smaller protective effect on the transition to ever smoker status for Latino than non-Latino youth (Table 2).

Table 3 shows the results of two nested logistic regression models with parent education as the independent variable and transition to current cigarette smoker as the dependent variables. Both models were estimated in the pooled sample. While *Model 1* only included the main effects of parent education, ethnicity, and covariates, *Model 2* also included four interaction terms between Latino and African American ethnicity and two levels of parent education.

**Table 3:** Logistic regressions on the transition to current cigarette smoker in the pooled sample.

	**Model 1**		**Model 2**	
	**Main effects**		**Main effects** **+** **interactions**	
	OR	95% CI	OR	95% CI
Ethnicity (Latino)	0.68^#^	0.46–1.03	0.28^**^	0.13–0.62
Ethnicity (African American)	0.24^***^	0.13–0.44	0.21^**^	0.07–0.64
Gender (male)	1.23	0.89–1.69	1.23	0.89–1.69
Age (18–21)	1.58	0.76–3.31	1.60	0.76–3.35
Parents married	0.54^***^	0.39–0.76	0.55^***^	0.39–0.77
**PARENT EDUCATION**				
Less than high school graduation	1.00		1.00	
High school graduation	0.94	0.61–1.45	0.58^#^	0.34–1.01
College graduation	0.63^#^	0.37–1.05	0.42^**^	0.23–0.76
High school graduation × Latino			3.64^**^	1.44–9.20
College graduation × Latino			2.43	0.58–10.16
High school graduation × African American				
College graduation × African American			1.04	0.27–4.01
Intercept	0.09		1.49	0.25–9.06

# *p < 0.1*,

* *p < 0.05*,

** *p < 0.01*,

*** *p < 0.001*.

*CI, confidence interval; OR, odds ratio*.

Based on *Model 1*, high parent education was associated with lower odds of transition to a current cigarette smoker. *Model 2* showed interactions between parent education and Latino ethnicity on the transition to a current cigarette smoker, which suggests a higher parent education has a smaller protective effect on youth tobacco use outcomes for Latino than non-Latino youth (Table 2).

## Discussion

The current study produced two findings. First, in the overall sample, high parent education was protective against youth transition to tobacco use. Second, the protective effect of high parental education against the eventual transition to cigarette smoking was diminished for Latino than for non-Latino youth. Thus, while parent education protects youth against transitioning to tobacco use, this protection is seemingly unequal, with ethnic minorities (i.e., Latino) gaining weaker protection than the majority (non-Latino). This is another example of how ethnicity, a marginalizing social identity, reduces the health return of a socioeconomic resource for a minority group.

The overall finding on protective effects of parental education against the transition of youth to tobacco outcomes is in line with what we know about social patterning of health, social determinants of health, fundamental cause theory, and social gradient in health. Extensive research in the U.S., Europe, and other parts of the world has established a protective effect of parental education as one of the main protective resources against the risk behaviors of youth. High parental education is not only protective against tobacco use it also protects youth against depression, anxiety, alcohol use, violence, and poor educational outcomes.

Our finding in Latino youth is in line with our previous work showing that high SES (e.g., educational attainment, income, marital status, and employment) African American youth and adults at a disproportionately high risk of substance use, a level which is not expected given their family SES (21, 23, 25, 52). This finding may also explain why high educated African Americans and Latinos remain at risk of asthma and chronic obstructive pulmonary disease (53). This might also be because highly educated Latinos and African Americans are exposed to a high level of tobacco advertisements, suggesting that they may be a target of predatory marketing strategies. Finally, it may be because African American and Latino youth report more peers and family members who use substances such as tobacco, and they attend schools that have a higher social-environmental risk that increases the risk of tobacco use.

These patterns are consistent with what we know about the impact of SES on other health outcomes such as substance use (25), anxiety (54), diet (55), exercise (56), depression (57), happiness (58), affect (58), suicide (59), self-rated health (60), obesity (11, 33), chronic disease (36), oral health (42), and mortality (61) are all smaller for African Americans than for non-Latino Whites. This study extends this literature to Latinos.

Besides, multiple analyses (9–11, 36, 37, 62) of 15 years of Fragile Families and Child Well-being Study (FFCWS) data have shown that high parental education and high family income at birth better protect obesity (11), grade point average (39), impulsivity (9), and ADHD (36) at age 15. The universal nature of the diminishing returns of family SES indicators on various health outcomes of non-Whites proposes that MDRs is a new, systematic, and understudied mechanism for transgenerational transmission of health inequalities in the United States. The FFCWS papers (9–11, 36, 37, 62) suggests that by the age of 15, parental education and family income have already generated unequal outcomes in youth (9–11, 36, 37, 62). Other research suggests that some of these inequalities occur before age 15 (33, 35). These findings have major implications for the elimination of health inequalities in the next generations. Interventions should happen across levels and before the age of 15.

At least some of these MDRs may be due to racial and ethnic residential segregation. Racial residential segregation will have a profound effect on the availability of cigarettes. There is a preponderance of advertising and the sale of cigarettes, especially loosies (single cigarettes) (63), in communities of color. Such sale (64) strategies allow for a more affordable cost of tobacco use in Latino and low-income communities who may not afford to purchase a pack of cigarettes (65). Greater education, however, does not mean that ethnic minorities can afford to purchase homes in neighborhoods where cigarettes and advertising are more regulated (13, 66–68).

Tobacco products are frequently advertised and promoted in the Latino communities (69, 70). Tobacco companies have historically tried to appeal to the Latino population through branding, financial contributions, targeted advertising, and other marketing strategies. For example, cigarette brands such as “Rio” and “Dorado” have been heavily advertised and marketed to the Latino community (69). As a result of these advertisements, these brands have appealed to the Latino-American community (69). The tobacco industry has also contributed to fund universities and colleges that support scholarship programs targeting Latinos (69). At the same time, the tobacco industry has funded Latino political organizations, cultural events, and the Latino art community (69).

There is a need to conduct more studies on the role of predatory marketing practices on ethnic and SES disparities in tobacco use. We argue that structural factors such as predatory marketing may generate MDRs on tobacco use of non-White youth. That is, predatory marketing and advertising may impose an additional risk of tobacco use to high SES Latino individuals through reducing the effects of SES. SES is shown to have smaller effects for Latino to bring them out of poor urban areas (68). High SES Latino youth may be exposed to a high density of tobacco retails and advertisements. Introducing more restrictive tobacco marketing regulations may be particularly beneficial to Latino populations. Example policies include banning additional point-of-sale advertisement, flavoring, coupons, and discounts in predominantly ethnic minority areas. However, all these hypotheses need additional research.

### Implications

The results may have some policy and public health implications. The results help increase “understanding [of] why people become susceptible to using tobacco products” (71). The results may extend the development of public policies that can be designed and implemented to address tobacco-related disparities such as more restrictive national and local marketing and other policies (72). Fortunately, the literature shows that currently, Americans tend to positively evaluate tobacco control regulations and do not view them as against their autonomy (72).

There is a need for both national and local policies that specifically address MDR-related disparities in tobacco use and related health conditions (7, 9, 11, 21, 25, 42, 52, 54, 73, 74). We need to study the roles of discounts, coupons, flavoring, and the density of tobacco retails in shaping MDR-related disparities in tobacco use (21, 25). Similarly, we need to study the role of tobacco control policies and regulations that may minimize MDR-related tobacco disparities. It is essential to explore how these marketing strategies target low SES Latino communities (21, 23, 25, 52). To undo the ethnic disparities in tobacco use, there is a need to stop predatory tobacco marketing in low-income areas. Minimizing MDR-related inequalities should be regarded as a core element of tobacco prevention strategies in middle-class ethnic minorities.

## Limitations

This study had some methodological limitations. The sample size was imbalanced across ethnic groups and tobacco use outcomes. As a result, and to avoid differential statistical power across groups, we did not conduct ethnic-specific models. Income, employment, marital status, and area-level SES factors were not included in this study. Parental smoking data could also be a confounder. Our study did not include any data on parental substance use. Geographic region and household size could also be relevant to both parental education and youth smoking. This study did not measure tobacco control policies that vary based on the geographic location of the participants. Our strengths included large sample size, random sampling, longitudinal design, and results that were generalizable to the U.S. Our longitudinal design allowed us to model the incidence of (transition to) smoking rather than the cross-sectional prevalence of tobacco use. Still, more research is needed on the mechanisms of MDRs of parent education on youth tobacco use.

## Conclusion

In the United States, minority youth are at a relative disadvantage compared to White youth in gaining tobacco use prevention benefits from their parent education. While higher parent education helped adolescents have lower tobacco use, this pattern is more pronounced in the most privileged (white) youth compared to Latino youth. As a result, we should expect a higher than expected risk of tobacco use in Latino youth from high SES families. Tobacco-related health disparities by ethnicity may be generated by factors beyond SES families. Research should explore structural factors that contribute to the reduced protective effects of parent education on tobacco use of middle-class Latino youth.

## Data Availability Statement

The datasets generated for this study are available on request to the corresponding author.

## Ethics Statement

All adolescent participants provided written assent. When they became adults, they provided consent. All the parents or guardians also provided permission and consent to be interviewed. The study protocol was revived and approved by the Westat Institutional Review Board. We, however, used the PATH public data set which is fully de-identified. As a result, the current analysis was non-human research and was exempt from an IRB review.

## Author Contributions

SA conceptualized the study, analyzed the data, prepared the first draft of the paper, and acquired the funding. MB and CC contributed to the revision and conceptualization of the study. All authors approved the final draft.

## Conflict of Interest

The authors declare that the research was conducted in the absence of any commercial or financial relationships that could be construed as a potential conflict of interest.
